# Research Progress in Membrane Lipid Metabolism and Molecular Mechanism in Peanut Cold Tolerance

**DOI:** 10.3389/fpls.2019.00838

**Published:** 2019-06-27

**Authors:** He Zhang, Jiale Dong, Xinhua Zhao, Yumei Zhang, Jingyao Ren, Liting Xing, Chunji Jiang, Xiaoguang Wang, Jing Wang, Shuli Zhao, Haiqiu Yu

**Affiliations:** ^1^Peanut Research Institute, College of Agronomy, Shenyang Agricultural University, Shenyang, China; ^2^College of Agronomy, Qingdao Agricultural University, Qingdao, China

**Keywords:** peanut, cold stress, membrane lipid metabolism, molecular mechanism, lipid signal transduction

## Abstract

Early sowing has been extensively used in high-latitude areas to avoid drought stress during sowing; however, cold damage has become the key limiting factor of early sowing. To relieve cold stress, plants develop a series of physiological and biochemical changes and sophisticated molecular regulatory mechanisms. The biomembrane is the barrier that protects cells from injury as well as the primary place for sensing cold signals. Chilling tolerance is closely related to the composition, structure, and metabolic process of membrane lipids. This review focuses on membrane lipid metabolism and its molecular mechanism, as well as lipid signal transduction in peanut (*Arachis hypogaea L.*) under cold stress to build a foundation for explicating lipid metabolism regulation patterns and physiological and molecular response mechanisms during cold stress and to promote the genetic improvement of peanut cold tolerance.

## Introduction

**FIGURE 1:**
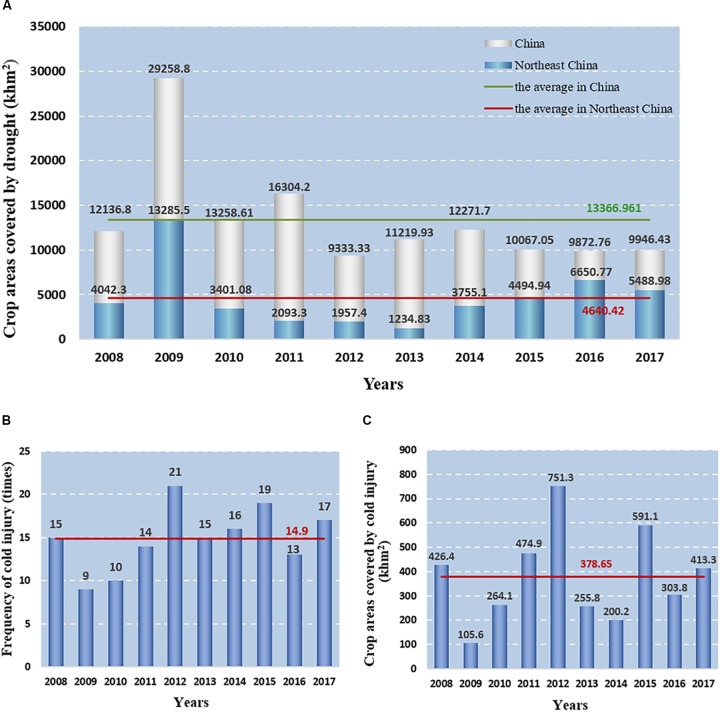
The occurrence of drought and low temperature disasters in China and Northeast China during 2008–2017. **(A)** The crop areas covered by drought in China and Northeast China. **(B)** Frequency of cold injury in Northeast China, and red line indicates the mean value. **(C)** The crop areas covered by cold injury in Northeast China, and red line indicates the mean value.

Peanut (*Arachis hypogaea L.*), one of the most important grain legumes as the source of edible oils and proteins, is cultivated in the semi-arid tropical and subtropical regions of the world (Katam et al., 2016). Recent statistics have shown that extreme weather events, particularly drought conditions caused by the changes in the global climate and water cycle, have occurred at an increasing frequency and intensity in peanut-producing countries, such as China and India (Yu et al., 2014; Lesk et al., 2016). In recent years, peanut planting areas have rapidly developed in high-latitude areas such as Northeast China. However, these regions are subjected to severe water-deficient conditions and seasonal drought, particularly from early May to the mid-May, the area covered by drought has been above 30% of the nation’s crop (Yang et al., 2018; Zhang et al., 2018; Figure 1A). According to the statistics of the Ministry of Water Resources of the People’s Republic of China (2018), the annual loss of industrial crops caused by drought in China accounts to 28.22 billion yuan, and peanuts account for about 20% (Li et al., 2014; Aninbon et al., 2016; Qin et al., 2017). Planting spring cultivars earlier is a feasible measure to circumvent spring sowing drought in peanut production, as well as in prolonging the vegetative growth period and increase nutrient accumulation for crop propagation (Rana et al., 2017). However, as a thermophilic crop, peanut needs relatively higher temperature throughout the whole development process (Wang et al., 2003). The lowest temperature of peanut germination is 12-15°C, and the peanut plant shows maximum growth at 28°C but experiences severe metabolic perturbations below 12°C (Bell et al., 1994). Sowing spring peanut earlier in Northeast China can impart deleterious effects on seed germination. In addition, chilling injury events have frequently occurred in Northeast China in the past few years (Ma et al., 2017; Shen et al., 2019; Figure 1B,C), severely influencing the peanut growth, development, bloom, and yield (Kakani et al., 2002; Wang et al., 2003; Chen et al., 2014; Table 1). Therefore, it is essential to optimize the comprehensive evaluation system of peanut cold tolerance and breed peanut germplasm with cold tolerance in Northeast China.

**Table 1:** Effects of cold stress on growth stages of peanut.

Growth stages	Optimal temperature (°C)	Minimum temperature (°C)	Symptoms of cold injury	References
Germination stage	25–37	12–15	Slow germination or even loss of the germination ability No seedling emergence in a large area in the field	Bell et al., 1994; Yan, 2001; Navarro et al., 2010; Chang et al., 2019
Seedling stage	25–35	14–16	Seedlings grow slowly or even stop growing Leaves dehydrated, wilted, yellowed and even withered to death Plant growth weakened	Awal and Ikeda, 2002; Prasad et al., 2006; Upadhyaya et al., 2009; Shi et al., 2009
Flower-pegging stage	25–28	22	Pollination hindrance Delayed flowering Decrease in the number of flowers Decrease in the number of needles	Song and Wang, 1979; Kakani et al., 2002; Wang et al., 2003; Liu et al., 2017;
Pod-setting stage	23–25	15	Pods develop slowly or even stop developing Decrease in the number of pods per plant Increase of empty shell rate	Song and Wang, 1979; Ketring, 1984; Nigam et al., 2010;
Pod-filling stage	23–27	4	Pods decay Seed mildew rate increased and inactivated	Ketring, 1984; Upadhyaya et al., 2009; Sorensen et al., 2009; Liu et al., 2017

Research studies on cold stress in plants have been conducted in the early 1830s, with a history of more than 180 years. Breeders have been trying to develop new varieties to resolve the problem of peanut chilling damage and have made certain progress, and a few cold-tolerance early-maturing cultivars with ability to germinate in cooler soils have been released (Ntare et al., 2001; Gorbet and Shokes, 2002; Upadhyaya et al., 2003, 2006). However, cold tolerance in plants is an intricate quantitative trait that always occurs in combination or in succession and it is not controlled by a single regulatory pathway or gene, making conventional breeding approaches for cold tolerance challenging (Kumar et al., 2015; Wang et al., 2017). With the development of biotechnology in agriculture, extensive and in-depth studies on the mechanism of cold tolerance in plants in terms of morphologicalanatomical, physiological, biochemical, and molecular biology have been conducted. Lyons (1973) proposed that chilling damage initially occurs at the cellular and organ levels. The biomembrane system, including cell membrane, nuclear membrane and organelle membrane, is the initial site of injury, particularly in terms of its structure, function, stability, and enzyme activity, thereby resulting in substantial metabolic imbalance, especially involving respiration and photosynthesis. These changes in turn affect the plant growth and development and eventually incur damages at the whole-plant level, leading to the occurrence of chilling damage. Biomembrane is also the main repository of lipid for peanut plants (Yu, 2008), and fatty acid is the main component of biomembrane, which has been used as the primary index to evaluate peanut quality. Recent studies have further shown that chilling tolerance in peanut is closely correlated with the composition and structure of the membrane lipids, particularly the saturation of membrane fatty acids (Tang, 2011). The complex physiological, biochemical, and molecular mechanisms between membrane lipid metabolism and cold tolerance is being continuously explored to improve cold tolerance by means of high-throughput gene identification, gene editing, and transgenic technology.

In this review, we summarize the effects of cold stress on membrane lipid metabolism, including permeability, peroxidation, component change, and unsaturation, as well as its molecular mechanism and lipid signal transduction in peanut under cold stress, to lay a foundation for the elucidation of lipid metabolism regulatory patterns and physiological and molecular response mechanisms in cold stress, as well as to promote the genetic improvement of peanut cold tolerance.

## Effects of Cold Stress on Membrane Permeability

**FIGURE 2:**
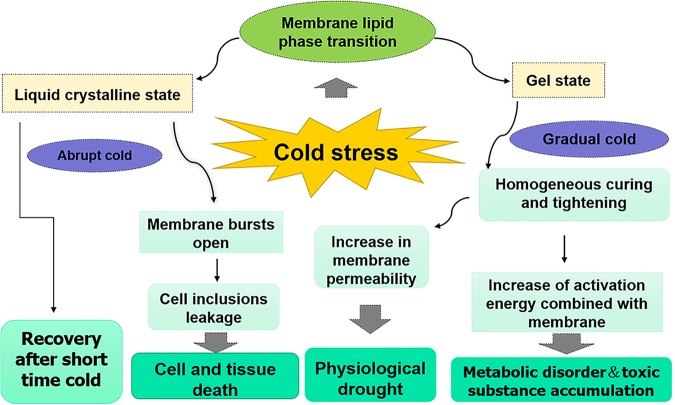
Effects of cold stress on membrane permeability. The main reason for the increase of membrane permeability in plants under cold stress is the phase transition of membrane lipids. When plants encounter an abrupt cold, membrane lipid is in liquid crystalline state, cold tolerant plants can recover in a short time, but the electrolyte leakage caused by membrane bursts open will happen in cold sensitive plants and ultimately lead to cell and tissue death. When plants encounter a gradual cold, membrane lipid is in gel state, the permeability of membrane increases with the prolongation of cold time, resulting in the loss of intracellular water and physiological drought. At the meantime, the increased activation energy of enzymes bound to membrane leads to metabolic disorder and toxic substance accumulation in plants.

The regulatory mechanism of biomembrane fluidity is one of the principal mechanisms that plants accommodate to changes in temperature conditions (Figure 2) and it is affected by the distribution ratio of various lipids on the membrane and the unsaturation of the glycerol lipid group (Li et al., 2016; Barrero-Sicilia et al., 2017). When peanut plants are subjected to cold stress, membrane lipids change from liquid crystal state to the gel state (Murata et al., 1982), which can cause the cessation of protoplast flow and an increase in membrane permeability, resulting in electrolyte leakage and loss of balance of intracellular ions (Huang et al., 2015). Chilling injury symptoms include dehydration, wilt, chlorosis, and accelerated senescence consequently happen (Upadhyaya et al., 2009). To maintain turgidity and original metabolic process, various organic and inorganic substances, such as inorganic salt, proline, betaine, soluble sugars, and soluble proteins, accumulate in plant cells via osmotic regulation, which lead to an increase in the concentration of cell fluid and a decrease in osmotic potential (Kishor and Sreenivasulu, 2014). Generally, under cold stress, proline, soluble sugars, and soluble proteins accumulate in the cytosol of sensitive and tolerant cultivars. Furthermore, the increasing amplitude of these osmotic regulation substances in varieties with stronger cold tolerance is larger than cold-sensitive varieties. However, the content of these significantly decreases in the cytosol when peanut plants are subjected to unbearable chilling (Bai D.M. et al., 2018; Kazemi-Shahandashti and Maali-Amiri, 2018).

Free proline accumulation is a heritable trait (Hanson et al., 1979) and can be used in screening genotypes for cold tolerance (Kim and Tai, 2011). Δ1-Pyrroline-5-carboxylate synthetase (P5CS) is a key enzyme in the glutamate pathway of proline biosynthesis. The overexpression of the *P5CS* gene in transgenic (introgressed with cDNA of the *P5CS* gene) plants results in increased cytoprotection and tolerance. The cDNA of the *P5CS* gene results in high levels of P5CS enzyme and a 10- to 18-fold increase in proline content, which contributes to both cold and drought tolerance through enhanced biomass production (Banavath et al., 2018). With the discovery of cDNA for *P5CS* and *P5CR* genes, future research could be directed at introgression of the gene, and the effect thereof, on yield and quality attributes of peanut under cold stress conditions in Northeast China.

## Effects of Cold Stress on Membrane Lipid Peroxidation

The damage of the plant biomembrane system at low temperature is also related to membrane lipid peroxidation and protein destruction induced by reactive oxygen species (ROS) (Grant et al., 2014). Membrane lipid peroxidation refers to a series of free radical reactions on double bonds of unsaturated fatty acid on the membrane, which is initiated by oxygen free radicals (O_2_^−^.^^, H_2_O_2_, ⋅OH) on unsaturated fatty acids in lipids (Thomas et al., 2016). ROS *in vivo* would produce abundantly and accumulate rapidly under cold stress that is far beyond the scavenging ability of antioxidant system, which breaks down the original equilibrium state of ROS. At this point, ROS begin to attack biological macromolecules, such as membrane lipids, nucleic acids, and proteins. The structure of the membrane system is also destroyed, resulting in a decrease in the photosynthetic rate, development of metabolic disorders, and massive accumulation of toxic substances in plants (Liu et al., 2013; Choudhury et al., 2016; Huang et al., 2016). Malondialdehyde (MDA) is the product of membrane lipid peroxidation that accumulates to higher concentrations in sensitive than tolerant genotypes (Iqbal et al., 2018a,b; Zhong et al., 2018). Cold-tolerant cultivars can resist external environmental stress by relying on the antioxidant enzyme system, which scavenges ROS and superoxide anion free radicals produced in plant cells, which mainly include superoxide dismutase (SOD), ascorbate peroxidase (APX), catalase (CAT), glutathione peroxidase (GPX), monodehydroascorbate reductase (MDHAR), dehydroascorbate reductase (DHAR), glutathione reductase (GR), and glutathione S-transferase (GST) (Tian et al., 2015).

Antioxidant enzymes are located among different sites of plant cells and work together with ROS-generating pathways to maintain ROS homeostasis (Zhang et al., 2015; Nejat and Mantri, 2017; Iqbal et al., 2019). The transcription factor APETALA2/ethylene response factor (AP2/ERF) plays an important regulatory role in signal transduction of the plant responses to various stresses including low temperature (Sakuma et al., 2002), which confers cold tolerance by promoting polyamine turnover, antioxidant protection, and proline accumulation. *ERF1*-Overexpressing plants have higher antioxidant activities, which are attributable to higher expression of genes, such as *Cu*, *Zn-SOD*, *CAT1*, *CAT2*, *CAT3*, and *cpAPX*, and accumulate more proline that is associated with induced *P5CS* and reduced *PROX2* transcription compared to the wild-type. These transgenic plants show reduced MDA contents, H_2_O_2_, and ROS accumulation under cold stress, which contribute to alleviating oxidative damage to biomembrane after cold stress treatment (Zhuo et al., 2018). To date, a variety of AP2/ERF transcription factors have been successfully identified and investigated in many plants, including Arabidopsis, rice (Nakano et al., 2006), wheat (Zhuang et al., 2011), soybean (Zhang et al., 2008) and rapeseed (Du et al., 2016). The peanut genome has eight *ERFs*, including *AhERF1–6*, *AhERF008*, and *AhERF019*. However, different expression patterns in relation to responses to abiotic stress have been described. For example, the expression of *AhERF4* and *AhERF6* is rapid and is substantially enhanced by abiotic stress, whereas the expression of *AhERF1* and *AhEERF5* are slightly enhanced under certain stress conditions (Chen et al., 2012; Wan et al., 2014). Interestingly, *AhDREB1* can improve tolerance to cold stress via the ABA-dependent pathway in Arabidopsis, and histone acetylation can affect the expression of *AhDREB1* under osmotic stress conditions, thereby improving plant cold tolerance (Bai H. et al., 2018).

## Effects of Cold Stress on Membrane Lipid Component

The membrane lipids of peanut plants are mainly composed of phospholipids (PL), which include phosphatidyl choline (PC), phosphatidyl ethanolamine (PE), phosphatidyl inositol (PI), phosphatidyl glycerol (PG), phosphatidic acid (PA), glycolipids (GLs) that consist of mono-galactose diglyceride (MGDG) and di-galactose diglyceride (DGDG), and a small amount of sulfolipids (SLs) and neutral lipids (NLs), such as cholesterol (Jouhet et al., 2004). Biomembrane is a dynamic equilibrium system that adaptively adjusts the internal composition based on changes in external temperature. Changes in lipid components are closely related to peanut abiotic stress, and the distribution ratio of lipids on the biomembrane of different tolerant cultivars will change with different degrees under various stresses (Lauriano et al., 2000; Sui et al., 2018). Phospholipids content is positively correlated to cold tolerance in plants, and cold tolerance is weakened when PL synthesis is blocked (Saita et al., 2016). Phosphatidyl glycerol is the main factor determining the membrane lipid phase transition for containing much saturated fatty acids, although it only accounts for 3–5% of thylakoid membrane lipids. The percentage of high-melting point molecules (C16:0/16:0 + C16:0/16:1t + C18:0/16:0 + C18:0/16:1t) in total molecular species or saturated fatty acids (C16:0 + C16:1t + C18:0) in total fatty acids in PG is significantly related to plant cold sensitivity, which is higher in cold-sensitive cultivars (Eriksson et al., 2011). The MGDG and DGDG are important components of thylakoid membrane lipids, which are closely related to photosynthesis, and their contents also change dynamically at low temperature (Kobayashi, 2016). Lipidomic analysis of maize leaves after cold treatment shows an increase in the PA and DGDG, but a decrease in PC and MGDG, resulting in enhanced turnover of PC to PA, which serves as precursors for galactolipid synthesis under low temperature conditions (Gu et al., 2017).

Lipid transfer protein (LTP) acts as a carrier for lipid transfer among different cell membranes. Changes in LTP activity can lead to alterations in membrane lipid composition and affect cold tolerance (Sun et al., 2015). Choi and Hwang (2015) reported that the *BLT101*-overexpressing transgenic wheat lines (BLT101ox) under cold stress loose less water and showed decreased expression of the genes induced by hormones (such as auxin and cytokinin) compared to non-transgenic (NT) plants. After prolonged cold treatment, BLT101ox leaves show normal phenotypes, whereas the NT plants displaydehydrated and withered leaves. Non-specific LTPs (nsLTPs), small molecular basic protein with abundant content, are responsible for the intermembrane transport of phospholipids by changing the composition of membrane lipids, participating in the biosynthesis of membranes, and transporting lipids among different organelles (Liu F. et al., 2015). There is also evidence that nsLTP is closely related to stress tolerance (Gangadhar et al., 2016). *LTP3* is positively regulated by the transcription factor *MYB96*, which mediates freezing and drought stress (Guo et al., 2013).

## Effects of Cold Stress on Membrane Lipid Unsaturation

Plants can modulate the stability and fluidity of membrane by changing the unsaturation of fatty acids in membrane lipids, which is of great significance for organisms to maintain normal photosynthesis and respiratory metabolism and resist cold stress (Mironov et al., 2012; Karabudak et al., 2014). In general, the content of unsaturated fatty acids in lipid membranes increases with decreasing temperature. In addition, compared to cold-sensitive cultivars, the content and the degree (number of double bonds) of unsaturated fatty acids in lipids are higher in cold-tolerant cultivars (Nejadsadeghi et al., 2015). The biomembrane of chilling sensitive genotypes undergoes a phase transition from liquid crystal to gel even at room temperature due to high saturation of fatty acids, whereas cold-tolerant genotypes can keep the phase transition temperature lower than the cold treatment temperature, thus avoiding phase transition (Ianutsevich et al., 2016). The main fatty acids in various peanut cultivars are similar, including palmitic acid (16:0), stearic acid (18:0), oleic acid (18:1), linoleic acid (18:2), linolenic acid (18:3), and arachidic acid (20:0). However, their contents vary among cultivars after low temperature treatment, i.e., contents of 18:1, 18:2, and 18:3 rapidly increase, whereas those of 16:0 and 18:0 decrease (Tang, 2011).

In plant cells, saturated fatty acids are synthesized by the type II fatty acid synthase system with the aid of an acyl carrier protein (ACP). The biosynthesis of unsaturated fatty acid is conducted by the desaturation of saturated fatty acids depending on two kinds of acyl lipases, which include glycerol-3-phosphateacyl transferase (GPAT) that is responsible for the lipidization on the C-1 position of the glycerol skeleton, and monoacyl-glycerol-3-phosphateacyl transferase (MGAT) that is responsible for the lipidization at the C-2 position, as well as various fatty acid desaturases (FAD) (Klempova et al., 2013). Acyl carrier protein is a small, acidic protein that plays an essential role in fatty acid synthesis by elongating fatty acid chains. In peanut, *AhACP1*, *AhmtACP3*, *AhACP4*, and *AhACP5* have been identified and have been proven to be closely linked with plant cold tolerance (Wei, 2012; Lei et al., 2014; Chi et al., 2017). The overexpression (OE) and antisense-inhibition (AT) of *AhACP1* in transgenic tobacco could alter the content of total lipids and composition of fatty acid in leaves, leading to a significant increase or decrease in the content of C18:2 and C18:3, thereby becoming more tolerant or sensitive to cold stress, respectively. It has been suggested that *AhACP1* bound to C18:1 might be the specific substrate of oleoyl-ACP thioesterase or GPAT and participate in membrane lipid synthesis (Yurchenko et al., 2014). The GPAT is the first acyl-lipidase in PG biosynthesis that can transfer the aliphatic acyl to C-1 position of glycerol 3-phosphate (G-3-P) to synthesize 1-acyl-glycerol-3-phosphoric acid (GPA). Cui et al. (2017) indicated that GPATs from different chilling-tolerant varieties have different selectivities to acyl group substrates, i.e., cold-sensitive genotypes prefer C16: 0, whereas chilling-tolerant genotypes have the same selectivity for C16:0 and C18:1. The expression of GPAT under cold stress is closely correlated to cold tolerance (Li et al., 2018). *AhGPAT3* and *AhGPAT5* are two genes that encode the GPAT protein, which plays a prominent role in the synthesis of peanut fatty acids, while its function in cold remains unclear (Hao et al., 2018).

## Fatty Acids of Membrane Lipids and Genetic Engineering of Cold Tolerance

**FIGURE 3:**
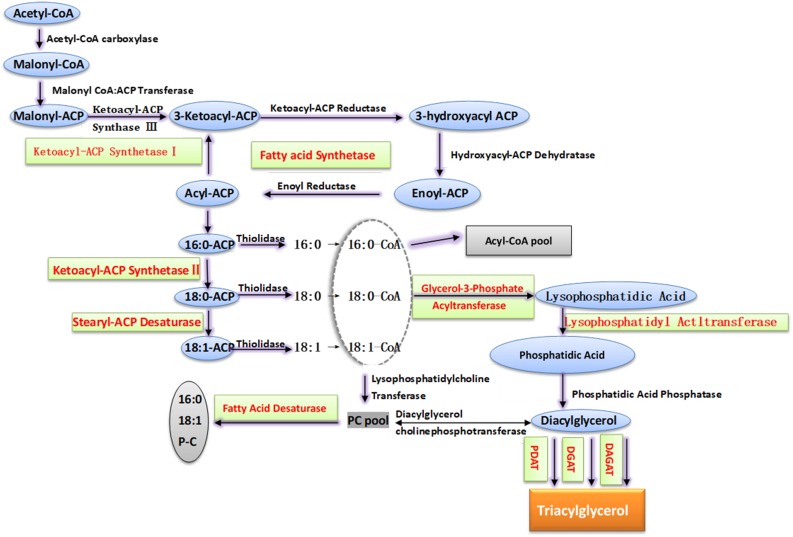
The pathway of membrane lipid biosynthesis in peanut. According to the substrate, the synthesis of triacylglycerol is divided into non-acyl-CoA dependent pathway and acyl-CoA dependent pathway. In the former, acyl groups transfer from phospholipid to diacylglycerol (DAG) to form triacylglycerol, and this step is catalyzed by phospholipid:diacylglycerol acyltransferase (PDAT). In the latter, glycerol-3-phosphate (G3P) is catalyzed by glycerol-3-phosphate acyltransferase (GPAT), lysophosphatidic acid acyltransferase (LPAAT) and diacylglycerol acyltransferase (DGAT) in turn and added the aliphatic group. The red characters in figure indicate the key genes in the lipid synthesis that have been identified and proved to play a vital role in peanut abiotic stress.

With the development of biotechnology, progress has been made in the genetic engineering of peanut cold tolerance. Several genes related to cold tolerance have been cloned and transferred to plants for functional studies (Cheng et al., 2013; Chen N. et al., 2016; Liu et al., 2016). The molecular regulatory mechanism under cold stress of fatty acid desaturation in membrane lipids includes regulating the expression of *FAD* to change the number of enzyme proteins, regulating the activity of FAD at post-translation level, and changing the available substrates to regulate the activity of FAD (Tovuu et al., 2016; Zhao et al., 2018). The fatty acid unsaturation of membrane lipid was mainly determined by the type and quantity of FAD, but few *FADs* in peanut have been functionally validated (Chi et al., 2011; Figure 3).

ω-3 FAD is considered the rate-limiting enzyme for the biosynthesis from diene fatty acids to triene fatty acids, and mainly responsible for catalyzing the introduction of the third double bond at ω-3 position. According to differences in subcellular localization, ω -3 FAD in higher plants can be divided into three types: FAD3 in the endoplasmic reticulum and FAD7 and FAD8 in plastids (Zhang et al., 2014). In Arabidopsis, *FAD3*, *FAD7*, and *FAD8* have been proven to mediate the synthesis of trienoic fatty acids from C18:2 and C16:2, and their expression enhance chilling tolerance (Chen et al., 2015; Roman et al., 2015). Interestingly, the structures of *FAD7* and *FAD8* are highly similar and thus have the same functions, while their enzyme activities vary in terms of responses to low temperature. No significant changes in triene fatty acid content were observed in the leaves of *fad7* mutant after cold treatment, and the expression of most *FAD7* genes is not affected by low temperature. Inversely, the *FAD8* gene is hardly expressed at normal temperature but induced by low temperature. It follows that *FAD7* is involved in plant growth and development under normal temperature, whereas *FAD8* participates in plant response to low temperature (Tang, 2007; Liu et al., 2014). The overexpression of *OsFAD8* substantially increases C16:3 and C18:3 content in leaves of transgenic rice lines, resulting in the damage of plant survival at 2°C for 7 days alleviated. The content of triene fatty acids in rice lines of *OsFAD8* silencing is reduced by 40.2% compared to that in the wild-type, although chilling tolerance decreased, allowing the plants to further adapt to a high-temperature environment (Nair et al., 2009). Wu et al. (2015) cloned a ω-3 Δ15- fatty acid dehydrogenase gene, *AhFAD3A*, which participates in the synthesis of α-C18:3 from cotyledons of germinated peanuts, and the expression of *AhFAD3A* is positively correlated with the formation of α -linolenic acid in peanut kernels and may be related to peanut tolerance.

The ω-6 FAD catalyzes monoenoic fatty acids to introduce the second double bond at ω-6 and form diene fatty acids, including FAD2 in endoplasmic reticulum and FAD4 and FAD6 in plastids (Park et al., 2016). Most of the known *ω-6 FAD* genes have multiple copies, and various copies of the same gene in the same plant vary in terms of the coding region, intron, and the length of the 5′UTR and 3′UTR (Cheng et al., 2013). *FAD2* gene family is functionally responsible for the conversion of C18:1 to C18:2 in peanut, and six novel full-length cDNA sequences (*AhFAD2-1*, *-2*, *-3*, *-4*, *-5*, and *-6*) have been identified. In addition, the *AhFAD2-1* gene is upregulated in developing seeds of peanut plants compared to the *AhFAD2-2* gene, while the *AhFAD2-2* gene is expressed most abundantly in the flowers, and they all play a major role in the conversion of oleic to linoleic acid (Wang et al., 2015; Wen et al., 2018). Accumulation of C16:1 and C18:1 in the *fad6* mutant of Arabidopsis thaliana results in a decrease in the level of polyunsaturated fatty acids in chloroplast membrane lipids and the number of thylakoids under cold stress.

Acyl-ACP desaturase is the only soluble desaturase family in peanut, including Δ9 stearyl ACP desaturase (SAD) and Δ 6 palmityl ACP desaturase (PAD). The SAD catalyzes the conversion of stearoyl-ACP to oleoyl-ACP and determines the properties of most cellular glycerol-lipids (Liu H.L. et al., 2015). Moreover, the *SAD* gene is induced by cold stress and enhances cold tolerance by increasing the enzyme activity and the unsaturated fatty acid content (Luo et al., 2014). *AhSAD3*, *AhSAD3A*, and *AhSAD3B* have been identified as possible target genes for manipulation of fatty acid saturation in peanut (Florin et al., 2011). Transgenic plants that overexpress the *SsSAD* gene exhibit significantly higher linoleic (18:2) and linolenic acid (18:3) content and advanced freezing tolerance (Peng et al., 2018). The expressions of *GhSAD2* gene in cotton plants after cold treatment at several levels were all upregulated; the level is highest after 6 h and then gradually decreases, thereby proving that the *GhSAD2* gene may play a vital role in the synthesis of unsaturated fatty acids in cottonseed oil. At the same time, it also plays a certain physiological role in cold tolerance (Cai et al., 2017).

## The Signal Transduction of Membrane Lipids Under Cold Stress

**FIGURE 4:**
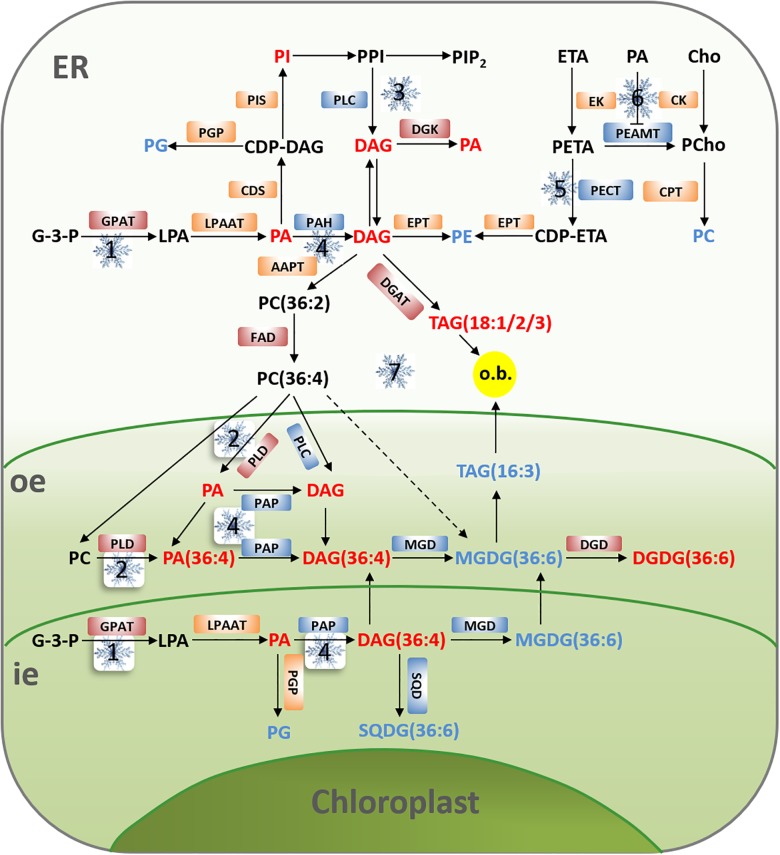
Model illustrating potential effects of cold stress on membrane lipid pathway in peanut. Under cold stress, there is an increase in the content of PA, PI, DAG, and DGDG (the red words), but a decrease in the content of PC, PE, PG, MGDG, and SQDG (the blue words). The main pathways to rapid cold-induced PA formation include the activity of GPAT is up regulated in the *de novo* biosynthesis of phospholipids (1), enhanced hydrolysis of PC by PLD resulted from the increase of PLD activity (2), the phosphorylation of PLC-generated DAG from PPI leads to DAG accumulation, which might cause an increased content of PA as a result from phosphorylation of DAG by DGK (3), or the inhibition of PAH/PAP activity by DAG (4). The activity of PECT is proposed to be down regulated by cold stress, and this would lead to reduced PE formation (5). As a second messenger, PA can inhibit the activity of PEAMT, thereby blocking the synthesis pathway of PC (6). During cold stress, the requirement for eukaryotic galactolipid biosynthesis is reduced and the activity of DGAT is upregulated, excess PC is converted to DAG and subsequently acylated to 18:1-, 18:2-, and 18:3-rich molecular species of TAG, which are contained in cytoplasmic oil bodies (o.b.). Simultaneously, turnover of MGDG in the chloroplast results in accumulation of low amounts of 16:3-containing, chloroplastic TAG (7). The red and blue boxes, respectively, represent the up-regulation and inhibition of the enzyme activities, the enzyme activities in orange boxes have no significant change or are not very clear yet before and after cold stress. AAPT, aminoalcohol phosphotransferase; CDP-ETA, cytidine diphosphate-ethanolamine; CDP-DAG, cytidine diphosphate-diacylglycerol; CDS, CDP-DAG synthase; Cho, choline; CK, choline kinase; CPT, phosphocholone cytidylyl transferase; EK, ethanolamine kinase; EPT, CDP-ethanolamine phosphotransferase; LPA, lysophosphatidic acid; LPAAT, lysophosphatidic acid acyltransferase; PAH, phosphatidate phosphohydrolase; PAP, phosphatidic acid phosphatase; PCho, phosphocholine; PEAMT, phosphoethanolaminemethyltranferase; PECT, phosphoethanolamine cytidylyl transferase; PETA, phosphoethanolamine; PGP, phosphatidic glycerol phosphatase; PIP2, phosphatidylinositol-4,5-bisphosphate; PIS, phosphatidylinositol synthase; SQD, sulfoquinovosyl diacylglycerol synthase; SQDG, sulfoquinovosyl diacylglycerol.

In the currently accepted model for temperature sensing, cold stress causes a change in membrane fluidity, and rearrangement of the cytoskeleton, followed by an influx of calcium that triggers downstream responses to confer cold tolerance(Guo and Liu, 2018). When cold signals are sensed by the plasma membrane, a series of signal transduction processes of membrane lipids are activated, leading to downstream actions to deliver the cold signal (Figure 4).

Phosphatidic acid is the precursor of PL biosynthesis, acts as the main lipid signal in eukaryotes, binds to specific protein, and activates the MAPK signal pathway, Ca^2+^-dependent protein kinase, NADPH oxidase, and ion channel (Testerink and Munnik, 2011). The biosynthesis of PA involves two different pathways: one is the direct hydrolysis of PL by phospholipase D (PLD), and the other way is phospholipase C (PLC) that catalyzes the hydrolysis of poly-phosphatidylethanolamine (PPI) together with diacylglycerol kinase (DGK) and synthesis PA from diacylglycerol (DAG) (Arisz et al., 2009). Extensive research suggests that PA is involved in the processes of plant growth, differentiation, reproduction, hormone response, and signal transduction under various biological and abiotic stresses (Hou et al., 2016; Meringer et al., 2016). Under cold stress, the two pathways of PLD/DGK and PLD are both responsive (Chen et al., 2015). Phospholipase D can catalyze the hydrolysis of phosphodiester bond and produce inositol triphosphate (IP3), diester glycerol (DAG), acetylcholine (Ach), and PA. As the second messengers in cells, IP3, DAG, PA, and Ach can cause a series of secondary reactions by changing intracellular Ca^2+^ and protein kinase K (PRK) levels, thus completing the process of cell response to cold signals (Hong et al., 2016). Moreover, the activity of PLD is closely related to the response of plant to low temperature (Muzi et al., 2016), short-term chilling stress (0–180 min) causes rapid and transient increases in PLD activity of young leaves, while long-term chilling stress (24–36 h) causes significant decreases in PLD activity in young leaves and roots (Peppino et al., 2017). Furthermore, PLD is also involved in ABA signal transduction under cold stress. ABA influences the activity of mitochondrial membrane – binding PLD in alpine ion mustard leaves through the mediation of Ca^2+^ under cold stress (He et al., 2017). As the initial enzyme of PL degradation, PLD can accelerate the degradation of PL, resulting in PA accumulation in the membrane. Phosphatidic acid can regulate the negative regulatory factor ABI1 of the ABA signaling pathway, as well as PLDal and PA that mediate ABA to induce upstream ROS accumulation and stomatal closure, thus contributing to chill tolerance in plants (Guo et al., 2012).

The MGDG and DGDG are vital components of chloroplast and thylakoid membrane lipids and are closely related to plant photosynthesis. They are synthesized by the catalysis of galactosylglycerol synthetase (MGD) and digalactoglycerol synthase (DGD), respectively (Rocha et al., 2018). Sensitive to feezing 2 (SFR2) is classified as a family I glycosyl hydrolase but has recently been shown to have galactosyltransferase (GAT) activity (Barnes et al., 2016). During freezing conditions, SFR2 transfers galactosyl from MGDG to another MGDG and produces oligogalactosols, including galactosyl diacylglycerol (GDG) and trigalactosyl diacylglycerol (TGD), leaving diacylglycerol (DAG) as a by-product (Roston et al., 2014). The DAG is converted into triacylglycerol (TAG), then TAG and oligogalactolipids derived from MGDG specifically increase in response to freezing (Vu et al., 2014). Therefore, the metabolic pathway of TAG is closely related to plant cold tolerance (Figure 3, 4). Diacylglycerol acyltransferase (DGAT) is a rate-limiting enzyme in the Kennedy pathway, one of the biosynthesis pathways of triacylglycerol (TAG) (Kennedy, 1963). In peanut, *AhDGAT1-1* and *AhDGAT1-2* heterologous expression in a Saccharomyces cerevisiae TAG-deficient quadruple mutant could restore lipid body formation, synthesis TAG and markedly accumulate higher levels of fatty acids (Peng et al., 2013; Tang et al., 2013; Chi et al., 2014). Recent studies have shown that DAGT1 plays a role in adaptive responses to chilling injury in plants, which can modulate the production of TAG and PA that cooperate with DGK (Chi et al., 2014; Chen B.B. et al., 2016; Yan et al., 2018). The expression of *DGAT1*, *DGK2*, *DGK3*, and *DGK5* in A. thaliana during cold stress can regulate the dynamic balance of DAG, TAG, and PA, thereby maintaining the integrity of membrane system and intracellular redox state (Tan et al., 2018). Furthermore, DGAT1 and SFR2 coexist in chloroplasts and the activity of DGAT1 may be necessary for the SFR2 pathway, and DGAT1 may improve cold tolerance by SFR2-mediated cold tolerance (Arisz et al., 2018).

## Conclusion and Prospective

Cold damage has become the key limiting factor of early sowing conducted to alleviate the spring sowing drought in peanut production in Northeast China. To cope with cold stress, plants have developed a series of physiological and biochemical changes and sophisticated molecular regulatory mechanisms, which display similarities and differences in various plant species. However, knowledge about physiological and molecular regulation mechanisms of peanut under cold stress in recent years has not been systematically documented. The plasma membrane is the barrier that protects the cell from injury and is also the primary place that senses cold signal. In the present review, we summarized the information on membrane lipid metabolism and its molecular response mechanisms, as well as lipid signal transduction in peanut under cold stress. Despite progress in elucidating the mechanism of cold tolerance in peanut, further investigations are warranted.

The cold signal is transduced from the extracellular to intracellular regions after being sensed by the plasma membrane and causes a series of physiological and biochemical changes. The ability to tolerate cold in peanut is based on the signal transduction by various factors in plant cells. However, how cold signals are perceived by plasma membranes and how cold signals transduce into intracellular through membranes are poorly understood. The diversity in plasma membrane composition, structure, and function is determined by the membrane lipids and membrane proteins. The interaction between membrane lipids and membrane proteins with different structures leads to differences in plasma membrane function. It is the key for analysis of cold signal transduction and elucidation of cold tolerance mechanism in peanut to understand the dynamic changes of plasma membrane structure and identify the function of the key protein.

The unsaturation of membrane lipids is closely related to cold tolerance in peanut. The proportion of unsaturated fatty acids has been regarded as an important index to measure the cold tolerance. Changing the ratio of saturated fatty acid to unsaturated fatty acid to improve cold tolerance peanut has become the research direction in recent years. However, the fatty acid composition of various lipids is variable, and it is not enough to analyze the fatty acid composition of membrane lipids in isolation to understand the physiological mechanism of membrane lipids. The main phospholipid molecules that make up the cell membrane and the main glycolipid molecules forming the chloroplast thylakoid membrane are also important in studying the physical phase transition of the membrane system at low temperature.

The development of emerging biotechnological methods in recent years, including CRISPR/Cas9, as well as the integration of omics and multi-omics, has impacted the agricultural sector by allowing the analysis of changes in lipid metabolism intermediates in the plasma membrane, the identification of differentially expressed genes related to lipids, and establishing a regulatory network for lipid metabolism under cold stress. This will be of great significance for how to satisfy plant growth requirements under deteriorating living conditions to sustain or even improve crop production.

## Author Contributions

HZ wrote the manuscript. JD, XZ, JR, LX, CJ, XW, JW, and SZ conceived the study. YZ provided valuable references and made great contributions to the later revision. HY revised the manuscript and gave final approval of the version to be published.

## Conflict of Interest Statement

The authors declare that the research was conducted in the absence of any commercial or financial relationships that could be construed as a potential conflict of interest.
